# The Origination of Life

**Published:** 1906-03-24

**Authors:** 


### The Hospital.

A Journal of

The Medical Sciences and Hospital Administration.

Vol. XXXIX.—No. 1,017. SATURDAY, MARCH 24, 1906.

### The Origination of Life.

The question of the possible origination of life upon this planet *de novo*, as distinguished from its transmission from ancestors, is one which has always had a quite peculiar power of exciting the emotions of philosophers, and of awakening an amount of partisanship not altogether favourable to the discovery of truth. In 1837, when Andrew Crosse observed the presence of living insects in immediate connection with his voltaic arrangements, and in metallic solutions supposed to be destructive to organic life, he was met, on publishing his discovery, to use his own words, "with so much virulence and abuse, in consequence of these experiments, that it seems as if it were a crime to have made them." It would presumably then have been possible, if modern methods and appliances had been available, to trace the examples of the *Acarus Crossii* to the ova from which they sprung; and, although the tracing was not actually accomplished, it is reasonable to believe that the commencements of life, if they could be brought within our ken, would be of a more simple character than highly developed insects. In 1872 Dr. Charlton Bastian published accounts of some experiments of his own which appeared to him to throw much doubt upon many received doctrines; and the hypotheses which he advanced were strenuously resisted by Tyndall, in a controversy which gradually became somewhat acrimonious, and ceased to be distinguished by the respect for the opinions of others which it is always decorous for men of science at least to profess to entertain. It was commonly held at the time that Dr. Bastian's views had been shown to be erroneous; and it is at least certain that Tyndall's experiments on sterilisation, and especially on the conditions necessary for the extinction of life in bacterial spores, have been of great practical value to subsequent investigators. For many years Dr. Bastian remained silent; but when, in 1897, he resigned his offices as a Professor in University College and Physician to University College Hospital, he determined to employ the leisure thus accruing to him in a resumption of his old investigations; and he has since published four "Parts" of "Studies in Heterogenesis," abundantly illustrated by micro-photographs. For a long period, however, he was met by an absolute refusal on the part of scientific contemporaries even to look at the evidence which he adduced; and the Dreyfus case itself was not more obstinately regarded as a *res judicata* than was the whole of the dispute which Dr. Bastian endeavoured to reopen. He quotes one philosopher who declared that he would not believe in the occurrence of certain transformations unless he could keep them under continuous observation, and who was not induced to depart from this view by the consideration that the transformations in question only occur in vessels secluded from the light.

Last week, however, Dr. Bastian assembled a large medical audience at the rooms of the Medical Society of London, and read an abstract of his observations, abundantly illustrated by micro-photographs projected upon a screen. Sir William Broadbent occupied the chair, and among others present were Sir Lauder Brunton, Dr. Pye Smith, Dr. Ferrier, Dr. Pavy, Dr. Buzzard, and many other distinguished physicians, while general science was represented by Sir Archibald Geikie. Starting from the point of view of his adversaries, to the effect that no bacteria or spores are contained in the cells of living and healthy vegetable tissues, Dr. Bastian first showed the development of bacteria and of monads within cells taken from the centre of a potato tuber, which had been soaked, prior to incision, in a solution of formaline, and preserved, with every possible care, from any external inoculation or contamination. He next showed a development of bacteria within the setæ of a water insect, and then passed on to the development of new forms from ova, as of a ciliated *Otostoma* from the egg of a *Hydatina*. He also showed the appearance of monads in zoogleal masses. The time was too short, and the subject too strange to the majority of the audience, to permit of any very fruitful discussion, and Sir Lauder Brunton and Dr. Pye Smith could do little more than admit the necessity for further research and for suspended judgment; while Sir Archibald Geikie, as a geologist, said that his chief difficulty arose from the shortness of the time within which the described developments or transformations had occurred. It is certain, we think, that Dr. Bastian has compelled the reopening of a question which had been regarded by many as a closed one, and that all the resources of modern science must be brought to bear in the directions which he has indicated. "The thing which has been," said

Solomon, "is that which shall be," and it seems not inconsistent with the order of Nature to suppose that her sources of vitality are inexhaustible, and that the reappearance of life upon the earth, if it were all extinguished, would be an occurrence in harmony with the laws under which it is now perpetuated and sustained. We entertain the highest respect for philosophic caution, but it is necessary to remember that caution may be unduly enhanced by prejudice, and that the brains even of philosophers may place physical obstacles in the way of new methods of thought.

